# Utilization of Electronic Health Records for Chronic Disease Surveillance: A Systematic Literature Review

**DOI:** 10.7759/cureus.37975

**Published:** 2023-04-22

**Authors:** Elina Guralnik

**Affiliations:** 1 Health Administration and Policy, Health Informatics, George Mason University, Fairfax, USA

**Keywords:** disease surveillance systems, public health informatics, chronic disease surveillance, public health surveillance, electronic health records

## Abstract

This study reviews the current utilization of electronic health records (EHRs) for chronic disease surveillance, discusses approaches that are used in obtaining EHR-derived disease prevalence estimates, and identifies health indicators that have been studied using EHR-based surveillance methods. PubMed was searched for relevant keywords: (electronic health records [Title/Abstract] AND surveillance [Title/Abstract]) OR (electronic medical records [Title/Abstract] AND surveillance [Title/Abstract]). Articles were assessed based on detailed inclusion and exclusion criteria and organized by common themes, as per the PRISMA review protocol. The study period was limited to 2015-2021 due to the wider adoption of EHR in the U.S. only since 2015. The review included only US studies and only those that focused on chronic disease surveillance. 17 studies were included in the review. The most common approaches the review identified focused on validating EHR-derived estimates against those from traditional national surveys. The most studied conditions were diabetes, obesity, and hypertension. The majority of reviewed studies demonstrated comparable prevalence estimates with traditional population health surveillance surveys. The most common approach for the estimation of chronic disease conditions was to use small-area estimation by geographic patterns, neighborhoods, or census tracts. The use of EHR-based surveillance systems for public health purposes is feasible, and the population health estimates appear comparable to those obtained through traditional surveillance surveys. The application of EHRs for public health surveillance appears promising and could offer a real-time alternative to traditional surveillance methods. A timely assessment of population health at local and regional levels would ensure a more targeted allocation of public health and healthcare resources as well as more effective intervention and prevention initiatives.

## Introduction and background

The wide adoption of electronic health records (EHR) in the United States in recent years presented an opportunity to consider additional use of the vast clinical data collected through EHR in population health surveillance [1-3]. To date, most population health surveillance is conducted through national surveys, such as the Behavioral Health and Risk Surveillance System (BRFSS) and the National Health and Nutrition Examination Survey (NHANES). A wide variety of EHR data has already been used in infectious disease surveillance [4]. However, the use of EHRs for chronic disease surveillance is less established and is a novel area of inquiry and knowledge.

In contrast to traditional surveillance methods, which involve self-reporting and a time lag in disseminating collected data and public health responses, EHR-based chronic disease surveillance presents two main advantages: timeliness and population-specific disease prevalence estimates that could inform locally relevant programs and interventions [5]. Currently, a few EHR-based surveillance systems already exist. It is yet to be established whether such systems would be able to provide reliable disease prevalence estimates at the state and local levels [6].

A recent study identified and classified challenges and solutions in the application of EHR to disease surveillance systems worldwide, with no specific focus on either infectious or chronic disease surveillance models [7]. The purpose of this study is to specifically examine the uptake of EHRs in chronic disease surveillance in the United States and explore the themes and patterns that have emerged so far in the approaches used and the health indicators studied.

The findings of this study were previously presented as a poster at the 2023 American Medical Informatics Association Informatics Summit on March 14, 2023.

Objectives

This study has three main objectives: (1) to identify some of the existing practices of EHR-based chronic disease surveillance nationwide; (2) to examine the approaches that are used within EHR-based surveillance systems or undertakings; and (3) to determine what chronic conditions have been studied through EHR-based surveillance methods.

## Review

Methods

This study followed the PRISMA review protocol; however, it has not yet been registered.

The eligibility criteria for this systematic review include an exhaustive list of inclusion and exclusion criteria. The eligibility criteria were strictly followed to provide an accurate assessment of the current uptake of EHR-based population health surveillance models for chronic conditions nationwide. Due to the fact that EHRs were not widely adopted until 2015, we chose to limit the study period to the years 2015-2021. Only U.S. studies were included in the final review (Table 1).

**Table 1:** Inclusion and exclusion criteria

Inclusion criteria	Exclusion criteria
Studies that report using EHR data for disease surveillance purposes	Studies with publication years prior to 2015
Studies that focus on chronic conditions only	Opinion articles that do not discuss practical applications
Studies that report non-communicable disease prevalence or incidence	Systematic reviews and/or conference proceedings
Studies that focused on feasibility of EHR-derived disease estimates and reported prevalence or incidence of chronic conditions	Studies that use EHR data to focus on risk of adverse events and disease progression
Studies that can be searched in databases: PubMed	Studies that focus on infectious disease surveillance
Studies that were published in academic journals only	Studies that do not report disease prevalence or incidence
Studies that were published in English language only	Studies or articles that focus on EHR use for assessing quality of care
Studies that were conducted in the US only	Studies that focus on drug safety surveillance using EHR
	Studies that focus on detecting occurrence of medical events with EHR data
	Studies that evaluate the effect of interventions with EHR data
	Studies that use EHR data for cancer screening or treatment needs

The data collection for this review occurred between March 16 and April 8, 2021. PubMed was the sole database used for the search.

Search

The PubMed database was searched using specific keywords that were broad enough to capture a full range of articles that would include data on EHRs and disease surveillance. Keywords used: (electronic health records [Title/Abstract] AND surveillance [Title/Abstract]) OR (electronic medical records [Title/Abstract] AND surveillance [Title/Abstract]). We applied two filters during the search - English language and study time, years 2015-2021. Our initial search returned 596 articles. Upon removing the duplicates, initial screening was applied. Articles were excluded based on the relevance of the topic, title, and abstract.

Screening by relevant titles/topics and abstract content

Screening involved removing articles that were systematic reviews, clearly identified as such, and opinion statements, clearly identified in the title. Screening by irrelevant titles - with infectious disease themes, a chronic disease without mention of EHRs or electronic medical records (EMR), or EHR data use other than for disease surveillance purposes. Examples of screening by irrelevant titles include assessing the quality of primary care, using EHR data for predictive modeling, or using EHRs as a source of data to answer research questions that are not relevant to population health surveillance. We identified 135 articles based on the initial screening process.

Further screening by relevant abstracts resulted in identifying initial patterns for data synthesis and organization: (1) EHR data to assess the prevalence of chronic conditions; (2) evaluation, development, and/or validation of a new or existing surveillance system; (3) EHR data repositories used for analyzing disease characteristics; and (4) evaluation of EHR primary care data for generalizability, sensitivity, and specificity of disease detection.

We identified 44 relevant articles to assess for full-text eligibility. The final screening was carefully grounded in our inclusion and exclusion criteria and identified only those studies that reported prevalence estimates. Studies that included the prevalence of disease patterns, the prevalence of common diagnostic categories, disease incidence estimates, and measures of concordance between EHR and another data source, all in the context of chronic disease surveillance, were also included [8-11]. We excluded all studies conducted outside of the U.S. The number of studies included for qualitative synthesis in the final review was 17 [5,6,8-25].

Data collection process

Data were independently extracted from the reports. Variables of interest in the review studies are shown in Table 2.

**Table 2:** Variables of interest

Variables	Description
Study author	Standard measure
Study location	To identify the uptake of EHR-based surveillance systems or studies that involve EHR-derived population health estimates in the US, nationwide
Study time period	Standard measure
Study sample size	Standard measure; to include a sample size of only one type of data source, EHR data, in the descriptive summary table (Table 3)
Study population	To identify which surveillance systems or studies specifically addressed children, adults, or both
Study institution	To identify the degree of partnership across different stakeholders in terms of data sharing, data quality, and system validation in the context of developing new EHR-based surveillance systems
Study outcome measure	Standard measures; to include (1) disease prevalence or incidence estimates and (2) measures of concordance between EHR-based and non-EHR data sources
Health indicators studied	The key measure of chronic conditions/indicators studied in the reviewed articles, including risk factor estimates associated with any known chronic conditions
Study design/approach	The key measure of interest in this study is to identify different approaches used in EHR-based disease surveillance
Data analysis method	The key measure of interest in the study is to identify analytical methods used in EHR-based surveillance as part of the approach used
The EHR-based data sources used	By type and name, to inform whether data are outpatient/inpatient and EHR system vendor name, if available
Non-EHR data sources used	By name and year, to inform which traditional surveillance survey was used in the study for concordance measures between EHR- and non-HER-based data sources
Principal summary measures	Studied design/approach, health indicator, and EHR-based data source used

**Table 3:** Description of included studies (N=17) *Only includes sample size of EHR-derived estimates.

Author	Study location	Study time period	Sample size*	Population studied	Study institution
Anthamatten et al. [12]	Denver, CO, USA	2013-2015	290,150 (children) 588,146 (adults)	Children and adults	Academic (University of Colorado), private and public (local and state health departments)
Chan et al. [13]	NYC, NY, USA	2011-2016	708,452	Adults only	Public (NYC DOH)
Davidson et al. [14]	Denver, CO, USA	2011-2012	21,578	Adults only	Public (Denver Public Health)
Figgatt et al. [15]	Philadelphia, PA, USA	2016	187,292	Children and adults	Public (Philadelphia DOH) and FQHCs
Filipp et al. [16]	Florida, USA	2012-2016	1,344,015	Adults only	Academic (University of Florida), private (PCORnet)
Flood et al. [17]	Wisconsin, USA	2011-2012	93,130	Children only	Academic (University of Wisconsin)
Horth et al. [18]	Utah, USA	2015	72,356	Adults only	Federal (CDC) and state (Utah DOH)
McVeigh et al. [5]	NYC, NY, USA	2013	716,076	Adults only	Public (NYC DOH)
Klompas et al. [6]	Massachusetts, USA	2012-2014	1,073,545	Adults only	Academic (Harvard), private and public (state health department)
Nichols et al. [10]	Multi-state (PA, WA, MN, MI, WI, NC, CO, CA, GA, OR, HI)	2005-2011	6,973,346	Adults only	Private (Kaiser Permanente), federal
Richardson et al. [19]	California, USA	2010-2014	412,400	Adults only	Private (Public Health Institute), public (California DOH)
Rowe et al. [8]	San Francisco, CA, USA	2015-2018	602	Adults only	Public (San Francisco DOH)
Sidebottom et al. [20]	Minnesota, USA	2006-2009	5,918	Adults only	Private (Allina Health)
Smith et al. [21]	Florida, USA	2012-2016	3.01 million	Adults only	Academic (University of Florida)
Turner and Keller [9]	Multi-state	2011-2014	730,785	Adults only	Academic (University of Virginia)
Wagaw et al. [11]	Chicago, IL, USA	09/2014-11/2014	527	Adults only	Public (Chicago DPH), federal (CDC, Alliance of Chicago Community Health Services)
Zhao et al. [22]	Wisconsin, USA	2007-2012	43,752	Children only	Academic (University of Wisconsin-Madison)

Risk of bias across studies

The studies included in the review may not be representative of all existing EHR-based chronic disease surveillance efforts that are ongoing, introducing publication bias. A few studies were retrospective by design, so there may be a time lag in reporting surveillance estimates or the results of validation measures that have been achieved.

Results

**Figure 1:**
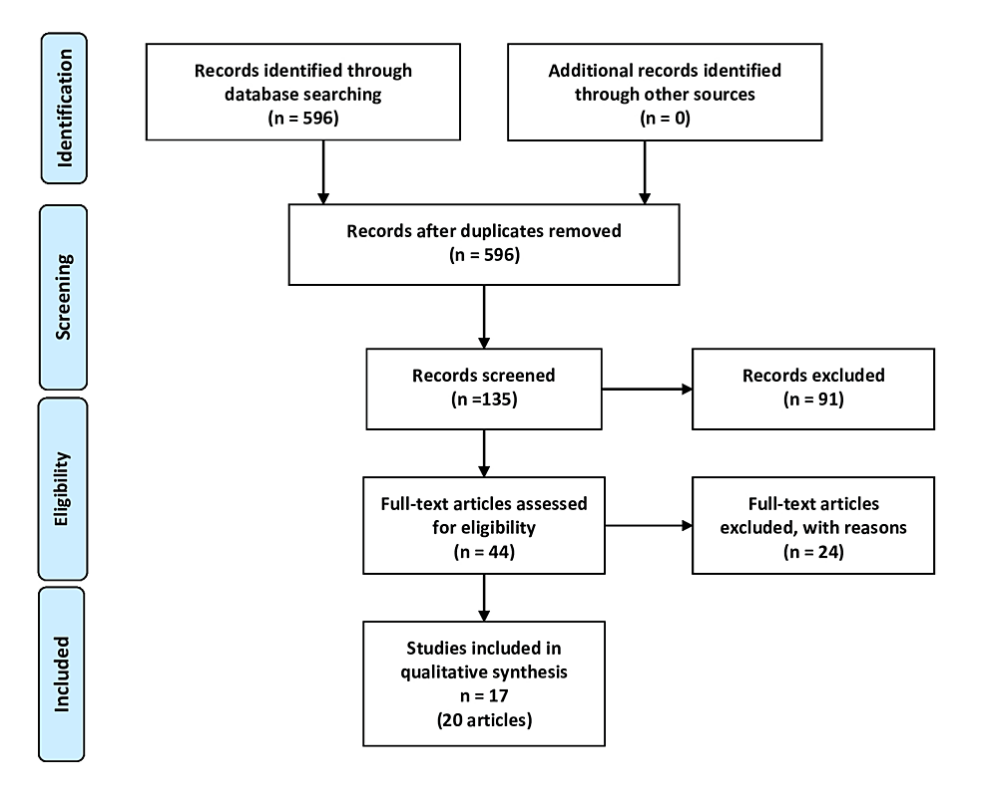
PRISMA flow diagram of the review process of EHR-based chronic disease surveillance

We identified 596 records after using relevant keywords for the search criteria. After initial screening, we selected 135 articles, which were further screened for relevance and US-only utilization of EHR-based chronic disease surveillance. From 44 articles that were screened based on full-text content, 20 articles were identified for final review (Figure 1). Of those articles, four were combined into one study as they were reporting estimates on the same health indicators from the same surveillance system [5,23-25].

Study characteristics

Table 4 presents the descriptive characteristics of the studies included in the final review. Seventeen unique studies have been identified that were feasibility or validation studies of an EHR-based chronic disease surveillance method/system. Two studies used multi-state EHR data, one of which focused on health estimates for a specific population, college students. Most study locations represented large urban or metropolitan areas. Most studies included multiple partnering organizations across academia, the public, private, and government sectors, with an overwhelming presence of academic institutions at the helm of those initiatives. Sample sizes varied across studies, with only two studies having a small sample size (<1,000 participants). The most studied populations were adults, with only four studies focusing on children’s disease prevalence estimates.

**Table 4:** Use of electronic health records for chronic disease surveillance. ^Only health indicators of interest were included in this table; *when no study design was specified or could be identified, EHR-based public health surveillance was used; **one of the three most common diagnostic categories in the college student population.

	Author	Study outcome measure	Health indicators studied^	Study design/approach*	Data analysis method	The EHR-based data source used (type and name)	Non-EHR data source used (name and year)
1	Anthamatten et al. [12]	Disease prevalence estimates, by geographic patterns	Obesity	EHR-based public health surveillance	Data aggregation by census tract, geocoding, and mapping	Multi-agency clinical data (Colorado BMI Monitoring System)	American Community Survey (2011-2015)
2	Chan et al. [13]	Disease prevalence estimates, by neighborhood	Hypertension, diabetes	Comparison of hypertension and diabetes estimates from EHRs and ED claims with survey-based estimates	Weighted prevalence estimates; Pearson correlation; choropleth maps (by prevalence quintile); absolute prevalence difference; absolute percentage-point difference;	Primary care-based system (Hub Population Health System)	ED claims-based (Statewide Planning and Research Cooperative System); NYC Community Health Survey (2012-2016)
3	Davidson et al. [14]	Disease prevalence estimates, by census tract	Depression	Survey-compared data sources for the ability to provide sub-county depression prevalence estimates.	Spatial analysis of depression prevalence; spatial rate smoothing combined with Queen Contiguity spatial weighting;	Common data model (the Colorado Health Observation Regional Data Service/CHORDS)	Denominator estimates – 5-year American Community Survey (2008-2012)
4	Figgatt et al. [15]	Disease prevalence estimates	(1) Obesity, (2) child asthma, (3) adult diabetes, (4) hypertension, (5) smoking	Calculation of prevalence of chronic disease (adult and children) and comparison of estimates with two population-based surveys with local estimates of disease prevalence	Chi-square analyses	Primary Care-based health center networks (Health Federation of Philadelphia and City Health Centers)	Behavioral Risk Factor Surveillance System (PA state data) and Public Health Management Company Household Health Survey
5	Filipp et al. [16]	Disease prevalence estimates, state-wide	Obesity	Comparison of EHR-based Florida obesity rates with rates obtained from the Behavioral Risk Factor Surveillance System	Level of agreement analysis: Bland–Altman plot	Clinical data research network consortium: OneFlorida Data Trust, part of National Patient-Centered Clinical Research Network (PCORnet)	Behavioral Risk Factor Surveillance System (BRFSS)
6	Flood et al. [17]	Disease prevalence estimates, by census block group, age, sex, race/ethnicity	Child obesity	Comparison of EHR-based child obesity rates to crude obesity rates and NHANES	Two-step weighting procedure (inverse probability weight and post-stratification correction)	Academic healthcare system/de-identified data repository for southcentral WI (Public Health Information Exchange/PHINEX)	National Health and Nutrition Examination Survey (NHANES) 2011-2012
7	Horth et al. [18]	Disease prevalence estimates	Diabetes, hypertension	Concordance of structured data (sex and age) and unstructured data (blood pressure reading and A1C); sensitivity and PPV for diabetes and hypertension in both data sources;	Iterative proportional fitting (raking); Linear regression models (prevalence as an outcome and regressors for ordinal age group); t-test for hypothesis testing	The health system of outpatient clinics; unstructured data from Clinical Health Information Exchange (outpatient, hospital, lab, Medicaid medication Hx)	American Community Survey (2015) marginal proportions for sex and 5-year age groups
8	McVeigh et al. [5]	Disease prevalence estimates, by geographic area	(1) Diabetes, (2) hyperlipidemia, (3) hypertension, (4) smoking, (5) obesity, and (6) depression	Comparison of EHR-based chronic disease estimates with non-EHR population survey estimates	two one-sided tests of equivalence (+/- 5 percentage point equivalence margin)	Ambulatory Care distributed data network (NYC Macroscope)	NYC HANES; Community Health Survey
9	Klompas et al. [6]	Disease prevalence estimates, by small-area estimates	(1) Diabetes (type 1 and 2 combined), (2) asthma, (3) smoking, (4) hypertension, and (5) obesity	Feasibility and comparability of prevalence estimates of chronic conditions from EHR-based and non-HER-based surveillance systems.	Pearson correlation coefficients for EHR-based crude and adjusted prevalence estimates versus BRFSS small-area estimates	Query-based distributed health data network (MDPHnet)	BRFSS and small-area estimates (CDC's 500 Cities data).
10	Nichols et al. [10]	Disease incidence estimates	Diabetes	Observational cohort analysis	Generalized linear model with a Poisson distribution; trends in diabetes incidence and glucose and HbA1c testing, overall and in prespecified groups	A multi-state consortium of integrated health-care delivery systems (Surveillance, Prevention, and Management of Diabetes Mellitus (SUPREME-DM) DataLink)	None
11	Richardson et al. [19]	Disease prevalence estimates, by income, race and ethnicity, and geography	Diabetes (type 2)	Validity of using HbA1c test results to determine diabetes prevalence. Estimation of disparities in diabetes prevalence by combining test results at a census-tract level	Sensitivity, specificity, positive predictive value (PPV), and the Pearson correlation coefficient for each HbA1c metric for the study population; geo-mapping	Private health system	US Census Data (2010); Diabetes Registry (Kaiser Permanente North Carolina)
12	Rowe et al. [8]	Prevalence of substance use patterns and ICD-10-CM codes	Substance use	Retrospective cohort	Sensitivity and specificity of using ICD-10-CM codes to detect substance use compared to retrospective self-report	Public healthcare system	Historical reconstruction interview data
13	Sidebottom et al. [20]	Risk factor estimates	CVD risk factors: BMI, BP, total cholesterol, smoking status	Retrospective cross-sectional	Two-sample t-tests for differences between the screening data and the EHR 1-year estimates; (2) Z tests for categorical measures;	Single integrated system (Allina Health EHR)	Community-wide CVD prevention initiative survey
14	Smith et al. [21]	Disease prevalence estimates, by geographic patterns, state-wide	Hypertension, uncontrolled blood pressure	Retrospective cross-sectional	Descriptive statistics - proportions by geographic distribution (county rates, by quintile); quantitative assessment of proof-of-concept	Clinical data research network consortium [OneFlorida Data Trust, part of National Patient-Centered Clinical Research Network (PCORnet)]	none
15	Turner and Keller [9]	Prevalence of most common diagnostic categories in the college student population	Mental health**	EHR-based population health surveillance	Descriptive statistics	Student health centers' EHR network (College Health Surveillance Network)	none
16	Wagaw et al. [11]	Measures of concordance between data from health survey and EHR for indicators contained in both data sources	BMI, weight, diabetes, high blood pressure, medication for high blood pressure, hyperlipidemia, tobacco use	Linking and validating self-reported data with EHR data of the same patients	χ² test for significant difference; Cohen’s k coefficient with 4 predefined agreement levels for concordance assessment	Primary care and safety-net clinics EHR	A self-administered web-based survey from a convenience sample (questions from Illinois BRFSS)
17	Zhao et al. [22]	Mean disease prevalence estimates, at the zip code level	Child obesity	Natural experiment	Generalized linear mixed model; small area estimation technique; non-parametric kernel smoothing for zip code missing/little data	Academic healthcare system/data repository for southcentral Wisconsin (Public Health Information Exchange/PHINEX); local community EHRs (Wisconsin Health Atlas/WHA)	None

Table 4 presents all the variables of interest for each reviewed study. We identified eight health indicators across all studies. The most common chronic diseases to have been reported with EHR-based disease prevalence estimations were obesity, diabetes, and hypertension. The two most common study approaches used were the validation of EHR-derived estimates with those from traditional surveillance surveys, such as the BRFSS or the NHANES, and the use of small-area estimation by geographic patterns, neighborhoods, or census tracts.

The most common limitation addressed in the studies was the sampling bias of EHR-derived population health estimates. The majority of the studies reviewed herein demonstrated comparable prevalence estimates between EHR-derived and traditional population surveillance surveys.

Discussion

Summary of Evidence

The utilization of EHR-based chronic disease surveillance is still nascent, with most studies conducting proof-of-concept, feasibility, or validation studies. The most common validation approach is to use estimates from EHR-derived data to compare them to those from traditional nationally representative surveillance surveys (i.e., NHANES, BRFFS, and ACS). This approach has shown comparable results across studies. There are only a small number (n=4) of studies that address disease prevalence estimates in children. Hence, there may be an opportunity and a need to develop more robust chronic disease surveillance models in the pediatric population.

The approach of small-area disease prevalence estimates with EHR-derived data is well recognized and shows promise in the estimation of local disease burdens and subsequent intervention programs and policy initiatives.

Obesity and diabetes, the most studied health indicators in both adults and children, suggest that there is a great need for timely prevalence estimates and emerging trends on the local level to determine the best path forward in addressing those chronic disease epidemics more effectively.

Also of note, only three studies have included both EHR and claims data to derive chronic condition prevalence estimates [13,16,21]. Two of those studies were part of the same clinical data research network, OneFlorida [16,21]. Given the fact that EHR data often presents as incomplete, it would be important to explore whether adding claims data to chronic disease surveillance systems would further improve the sensitivity of such systems and their concordance with traditional surveillance survey estimates.

Limitations

This study may not have been able to identify all EHR-based surveillance systems or models currently in use or development in the US due to a lag in reporting and inclusion criteria only focusing on the last five to six years of available data.

## Conclusions

The use of detailed clinical data from electronic health records for chronic disease surveillance is still limited but is gaining traction. Future studies are needed to assess whether EHR-based chronic disease surveillance can augment or entirely replace current estimates derived using traditional surveillance methods, such as national surveys, in the coming years. To date, national surveys remain problematic due to the time lag between data collection and the dissemination of relevant prevalence estimates. Based on the reviewed surveillance systems, the estimates derived from the electronic health records for population health surveillance appear to be comparable to those from the national surveys while promising a timely assessment of population health and a more targeted allocation of public health resources.
